# Implications of intracochlear decomposition gas formation in non-putrefied cadavers

**DOI:** 10.3389/fsurg.2024.1365535

**Published:** 2024-06-14

**Authors:** Philipp Mittmann, Arne Ernst, Rainer Seidl, Gina Lauer, Leonie Gölz, Sven Mutze, Marc Windgassen, Claas Buschmann

**Affiliations:** ^1^ Department of ENT, Unfallkrankenhaus Berlin, Berlin, Germany; ^2^ Department of Radiology and Neuroradiology, Unfallkrankenhaus Berlin, Berlin, Germany; ^3^ Institute of Legal Medicine and Forensic Sciences, Charité University Medicine Berlin, Berlin, Germany; ^4^ Institute of Legal Medicine and Forensic Sciences, University of Kiel, Kiel, Germany

**Keywords:** postmortem CT, intracochlear gas, hanging, radiology, decomposition gas

## Abstract

**Introduction:**

Postmortem computed tomography (pmCT) prior to forensic autopsy has become increasingly important in recent decades, especially in forensic documentation of single injuries, injury patterns, and causes of death. Postmortem decomposition gas formation can also be detected in pmCT scans, which might affect cochlear implant research in postmortem human temporal bones (TBs).

**Material and methods:**

Fifty non-putrefied hanging fatalities within a 2-year period (January 2017 to December 2019) were included with 100 TBs. Each body underwent whole-body pmCT prior to forensic autopsy. PmCT scans were analyzed with respect to the presence of intracochlear gas despite the lack of putrefaction at autopsy by an experienced fellow neurotologist.

**Results:**

PmCT revealed gas formation in two individuals despite the lack of head trauma and putrefaction at postmortem examination and autopsy. Both individuals showed enclosed gas in the vestibule and the cochlea on both sides.

**Discussion:**

Intracochlear gas formation, most likely related to decomposition, may occur despite the lack of putrefaction at postmortem examination and autopsy and can be detected by pmCT. This finding seems to be rather rare in non-traumatic death cases but might affect cochlear pressure research in postmortem human TB.

## Introduction

Postmortem computed tomography (pmCT) provides additional information prior to forensic autopsy (1). With high-resolution multi-slice computed tomography in a short, non-invasive, reproducible examination, dimension and location, especially of gas formation within the body, can be determined, i.e., vascular gas embolism can be illustrated in a 3D reconstruction using volume rendering (2).

After cardiac arrest, cellular aerobic metabolism changes into anaerobiosis due to the lack of oxygen. First, metabolism switches into glycolysis, but after consuming all glucose, cellular metabolism comes to rest. Mechanisms that protect cells from autolysis cannot be preserved, and hydrolytic enzymes are released. High concentrations of these enzymes are found in the gastrointestinal tract. Thus, visible putrefaction signs are usually first seen at postmortem examination in the lower right part of the abdomen (greenish skin discoloration), usually beginning 1–2 days after death at room temperature (20°C). This might vary if different areas of the body are significantly exposed to higher temperatures than other areas (body found with head in front of a heater, etc.). Intracochlear decomposition gas formation has not been described yet.

Conventional autopsy proof of intracochlear gas formation is impossible but has a major impact from a clinical point of view as it can lead to vertigo (3) or hearing loss (4). High-resolution computed tomography is the gold standard for detecting intracochlear gas in living individuals (3).

Intracochlear gas is also relevant regarding research about intracochlear pressure changes either induced by sound or evoked by cochlear implant surgery. The matter of ongoing research, in terms of preservation of residual hearing, is to keep intracochlear pressure changes during cochlear implantation low (5–7). Recent intracochlear pressure (ICP) experiments used artificial cochlear models, e.g., an artificial transparent cochlear model as in our setting. If such air bubbles occur, they need to be removed since they interfere decisively with the recordings otherwise (8). In contrast to artificial cochlear models, “real” human temporal bones (TBs) are subject to cochlear implant research. Nevertheless, transferring the results known from the model into the human bone is interesting and challenging. Different study groups have already proposed intracochlear pressure changes in the temporal bone model (9–11). As known from the model experiments, enclosed intracochlear gas can bias results (8); therefore, strategies are needed to identify enclosed gas in human temporal bones for research purposes. Most groups use laser Doppler vibrometry prior to intracochlear pressure measurements to verify the absence of enclosed gas (10, 12) in human temporal bone experiments. Nakajima et al. measured the round window velocity at a low frequency [400 Hz, 110 dB sound pressure level (SPL)] to verify the opposite (180°) motion relative to the stapes to rule out the presence of gas in the human cochlea (12). However, laser Doppler vibrometry requires logistical effort and extra time (10). Thus, it is reasonable to search for alternative approaches, i.e., if the air bubbles could be detected radiologically. The radiological quality of the pmCT scans has been shown to be sufficiently well suited for this purpose when there exists stable cooperation between clinicians and forensic pathologists (13). To date, to our knowledge, postmortem intracochlear gas due to putrefaction has not been described (14) but is essential in experiments with human temporal bones regarding intracochlear pressure changes (10, 15).

The aim of this study was to evaluate whether intracochlear decomposition gas formation can be detected by pmCT.

## Materials and methods

The study was approved by the institutional review board (IRB-ukb-HNO-2022/10) and conducted according to the principles expressed in the Declaration of Helsinki.

Fifty death cases within a 2-year period (January 2017 to December 2019) were included in this prospective autopsy study. In all the bodies, both TBs could be included (*n* = 100) and the postmortem interval could be determined. As the exact time of death was often unknown due to unwitnessed death, the minimal postmortal interval (m-pmi), i.e., the minimum time between the finding of the body and the autopsy, was calculated. Inclusion criteria were death by hanging in the absence of putrefaction at postmortem examination and autopsy, any known head trauma or cranial surgery prior to death, and a completed pmCT scan prior to autopsy. Trauma to the temporal bone immediately before death could be out ruled in every patient. Each body was scanned using a 16-slice CT scanner (Toshiba Activion; Toshiba Medical Systems GmbH, Neuss, Germany) with a slice thickness of 0.5 mm and an overlap of 0.3 mm. Reconstruction of 2D and 3D volume-rendered images was performed as described previously (16, 17). CT images were analyzed, and the results were interpreted by an experienced neurotologist with experience in the use of diagnostic imaging in a forensic setting and an experienced neuroradiologist.

## Results

**Figure 1:**
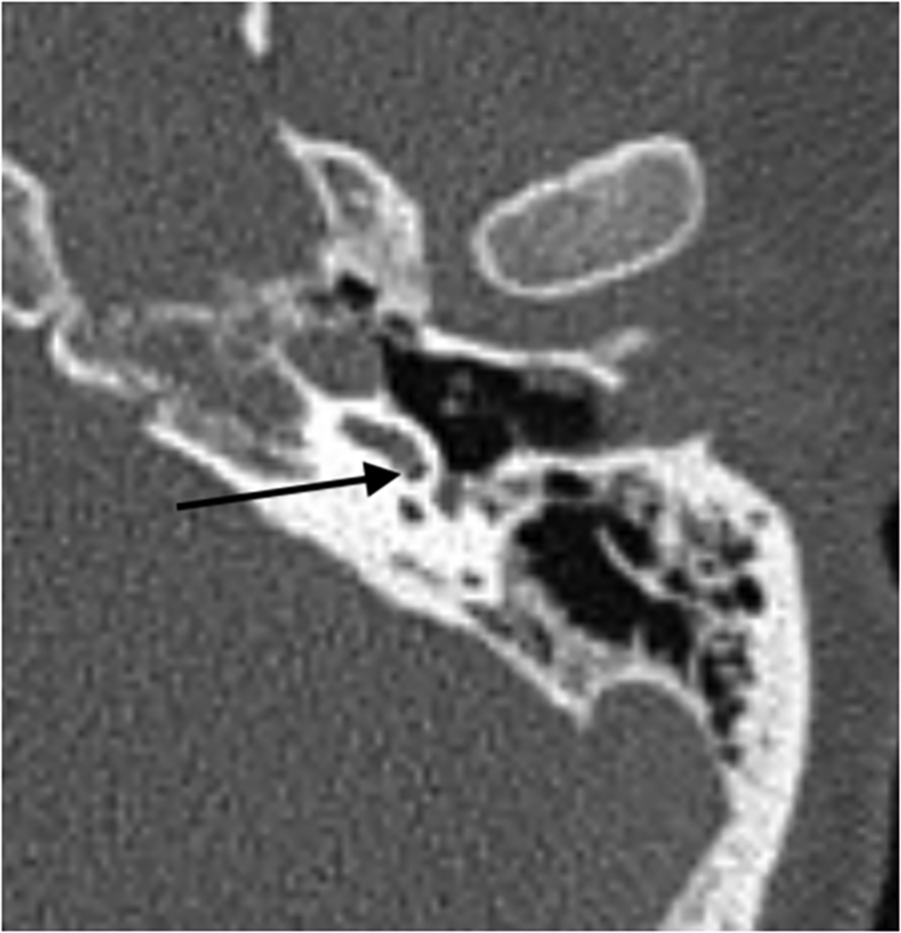
Axial pmCT scan. The left side with a gas bubble in the basal turn of the cochlea close to the round window (m-pmi 169.5 h). The arrow points to the bubble.

There were 39 male bodies and 11 female bodies, with a mean age of 45 years (range 18–96 years). The cause of death was hanging in all cases. The mean m-pmi was 157.21 h, ranging from 85.5 to 253.5 h. Forensic autopsy revealed no signs of visible putrefaction, trauma, or any kind of cranial surgery prior to death. Enclosed gas was found in two temporal bones by pmCT. On the left side, in one case, a single gas bubble was detected in the basal turn of the cochlea close to the round window (m-pmi 169.5 h, Figure 1).

**Figure 2:**
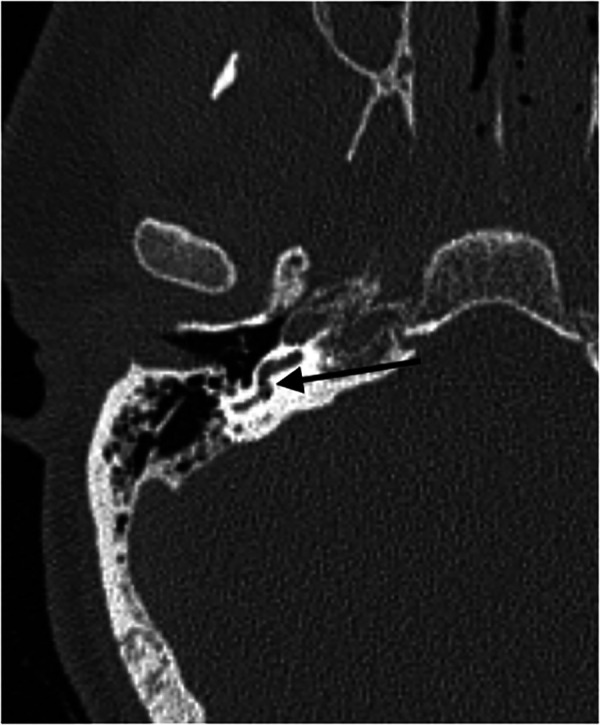
Axial pmCT scan. The right side with two enclosed gas bubbles in the basal turn of the cochlear and in the labyrinth (m-pmi 111 h). The arrow points to the bubble.

In the second body, two enclosed gas bubbles were found, one in the basal turn of the cochlear and one in the labyrinth (m-pmi 111 h, Figure 2). All other pmCT scans showed no sign of intracochlear gas. In summary, 2% of the investigated temporal bones showed enclosed gas in the cochlea and labyrinth complex.

## Discussion

Intracochlear and intralabyrinthine gas was detected in 2 out of 100 investigated temporal bones. As trauma or any kind of cranial surgery was not present, formation of decomposition gas is the only remaining explanation for this finding. Decomposition gas is well known to occur first in the abdominal cavity 1–2 days after death (18, 19). In contrast to other parts of the human body, such as the heart or abdomen, intracochlear decomposition gas cannot be aspired or even detected without postmortem computed tomography (20).

With increasing postmortem interval, putrefaction will arise, and the amount of decomposition gas within the body will also increase (20), whereas icing after death leads to lower density in postmortem CT (21). In our population, the postmortem interval ranged from 85.5 to 253.5 h, but intracochlear decomposition gas was only found in two individuals. The statistical calculation for two positive cases clearly forbids themselves.

PmCT is a valuable method to detect decomposition gas within different parts of the body (2). The proof of intracochlear decomposition gas in this population of non-traumatic deaths has never been described before. Although the amount of gas within the cochlear and the labyrinth is rather small, it most likely has reached its final destination via the connecting routes to the cerebrospinal fluid (CSF) space. The vestibular and/or cochlear aqueduct are those pathways. However, after death and the onset of anaerobic metabolism, the ducts themselves might be plugged (clogged) meaning gas cannot pass. Second, as the number of cells within the cochlea and the vestibule is very small, anaerobiosis and metabolism can only include a small number of cells within a small voluminal space. Due to the minimal volume of enclosed intracochlear and intralabyrinthine air, the enclosed amount cannot be quantified.

Intracochlear gas influences the quality of life in humans. Introduction of air into the scala tympani of the cochlea causes a decrease in cochlear potentials (22). Air perfusion in the scala vestibuli decreased cochlear potentials more drastically than in the scala tympani (22). Karimi et al. found gas enclosed in the cochlear in a patient after climbing a mountain (3). This patient underwent cochlear implantation before, and it can be assumed that gas got into the cochlea via cochleostomy due to the air-pressure changes (3). From a forensic point of view, detection of intracochlear gas without head trauma can aid in reconstructing circumstances of death, i.e., possible vertigo. Although differentiation of postmortem intracochlear decomposition gas and premortem traumatic intracochlear gas cannot be established conventionally or radiologically, the presence of intracochlear gas could be crucial regarding forensic reconstruction of the circumstances of death.

In studies on intracochlear pressure changes during cochlea implantation, one should bear in mind that enclosed intracochlear air distorts the results (8). Many studies come from artificial cochlear models; these are transparent, and intracochlear gas can be identified visually (5–7). However, this is not the case in temporal bone experiments (9–11). An indirect proof of the absence of intracochlear air can be done by laser Doppler vibrometry (10, 12); however, today, direct proof of the absence of postmortem intracochlear gas does not exist. With the knowledge that putrefaction and formation of decomposition gas within the cochlear is rare, temporal bones removed postmortem could be used as a proper model for intracochlear pressure changes in cochlear implantation research. This underlines research in the field and the recently published work from different groups (11, 15).

## Conclusion

Intracochlear decomposition gas is a rare finding in non-traumatic deaths. Nevertheless, this finding is important in research in the field of intracochlear pressure changes during sound experiments or intracochlear manipulation (e.g., cochlear implantation). We recommend pmCT before experiments in “real” human temporal bones to exclude intracochlear enclosed gas, i.e., decomposition gas formation even in non-putrefied bodies.

## Data Availability

The raw data supporting the conclusions of this article will be made available by the authors without undue reservation.
